# Predictive Factors of Significant Findings on Capsule Endoscopy in Patients with Suspected Small Bowel Bleeding

**DOI:** 10.3390/diagnostics14212352

**Published:** 2024-10-22

**Authors:** Ali A. Alali, Reem Alrashidi, Farah Allahow, Abhijit Dangi, Ahmad Alfadhli

**Affiliations:** 1 Department of Medicine, Faculty of Medicine, Kuwait University, Jabriyah 13110, Kuwait; 2 Thunayan Alghanim Gastroenterology Center, Amiri Hospital, Sharq 15300, Kuwait; 3 Haya Al-Habeeb Gastroenterology Center, Mubarak Alkabeer Hospital, Jabriyah 13110, Kuwait

**Keywords:** gastrointestinal bleeding, obscure gastrointestinal bleeding, capsule endoscopy, inflammatory bowel disease, small bowel

## Abstract

**Background:** Small bowel capsule endoscopy (SBCE) is an established non-invasive diagnostic modality for a variety of small bowel pathologies and has a significant role in altering the treatment course. The diagnostic yield of SBCE in the published literature varies widely between 45 and 75%. Furthermore, it is unclear if any patient-related factors predict higher diagnostic yield. The aim of this study is to report the diagnostic yield of SBCE for suspected small bowel disease and identify any predictive factors for identifying significant pathology on SBCE. **Method:** A retrospective study was conducted at Mubarak Al-Kabeer Hospital in Kuwait for patients who underwent SBCE between October 2013 and February 2022. All patients underwent upper and lower endoscopy prior to referral for SBCE. Patients’ medical records were reviewed to determine SBCE indications, results, and complications. The significance of the SBCE finding was classified according to the Saurin system. A logistic regression was performed to characterize baseline predictors for identifying significant pathology on SBCE. **Results:** Overall, 210 patients underwent SBCE and were included in the analysis. The mean age was 57.9 years (SD 18.5), and 129 (61.4%) were males. The most common indication for SBCE was obscure occult gastrointestinal bleed (75.7%), obscure overt gastrointestinal bleed (28.6%), and investigating gastrointestinal symptoms (7.6%). Adequate bowel preparation was achieved in most patients (88.1%), imaging of the entire small bowel was achieved in 194 patients (92.4%), and no adverse events were recorded. The overall diagnostic yield of SBCE for small bowel disease was 68.1%. The most common findings were vascular lesions in the small bowel (40.0%), small bowel ulcers (22.9%), and erosions (22.9%). On multivariate regression analysis, melena at baseline was significantly associated with increased odds of identifying high-risk lesions (Saurin class P2) (OR 2.1, 95%CI 1.03–4.30, *p* = 0.04). **Conclusions:** SBCE is an effective and safe tool for investigating small bowel pathology with a diagnostic yield of 68.1% in carefully selected patients undergoing such a test. Melena at baseline is the strongest predictor of identifying high-risk lesions, and patients with which should be prioritized for SBCE.

## 1. Introduction

Small bowel diseases pose a significant challenge in clinical practice due to the heterogeneity of the pathologies and the difficulties in visualizing this part of the gastrointestinal (GI) tract using conventional endoscopy [1]. Common small bowel diseases include Crohn’s disease, celiac disease, tumors, and vascular lesions, which can result in a variety of symptoms, including GI bleeding and obstruction [2]. Small bowel bleeding is an important cause of obscure gastrointestinal bleeding (OGIB) where the etiology of the bleeding is not identified after initial upper and lower endoscopic examination [3]. Even though small bowel bleeding accounts for approximately 5% of all GI bleeding, it exerts a significant burden on healthcare systems since many patients with which consume medical resources and require numerous investigations to try to identify the source of such bleeding [4]. Furthermore, the quality of life of affected patients is greatly reduced due to frequent hospitalizations, in addition to being subjected to multiple invasive procedures to try to identify the source of the bleeding [5].

The introduction of small bowel capsule endoscopy (SBCE) more than 20 years ago helped to revolutionize the assessment of small bowel pathologies by providing a non-invasive means to assess the small bowel [6]. Several studies have demonstrated the superiority of SBCE over radiological testing, including small bowel follow-through and CT for the detection of small bowel bleeding [7]. In addition, SBCE had a similar diagnostic accuracy to device-assisted endoscopy while providing the advantage of being a non-invasive test [8]. Nevertheless, the reported diagnostic accuracy of SBCE varies widely in the literature between 45 and 75% [9,10,11]. This variation may be related to the heterogeneity of the included patients and indications for testing, and a more accurate and selective performance of this test may improve the diagnostic yield while reducing the cost of unnecessary testing in low-yield indications. However, the subgroup of patients with the highest diagnostic yield remains poorly characterized in the published literature. The aim of this study is to evaluate the diagnostic yield of small bowel capsule endoscopy in patients with suspected small bowel disease and try to identify baseline factors associated with higher diagnostic yield among tested patients.

## 2. Methods

### 2.1. Study Design

A retrospective chart review was conducted, where all patients who underwent SBCE at Mubarak Alkabeer Hospital in Jabriya, Kuwait, between October 2013 and February 2022 were identified. The capsule endoscopy report was retrieved, and any abnormal findings were confirmed by reviewing the capsule images and video. Relevant clinical patient information was collected from the hospital electronic computer system, including patient demographics, clinical data (including symptoms, laboratory results, and follow-up data), imaging findings (including imaging modality and presence of gastrointestinal mass or inflammation), and endoscopic data, including previous endoscopic evaluations and any further endoscopic intervention performed after the SBCE. The SBCE data were recorded including the presence and type of any gastrointestinal pathology, adequacy of bowel preparation, capsule retention, and any complications related to SBCE. Patients were included in the study if they were at least 18 years of age and had sufficient clinical data documented in their chart. Ethical approval was obtained from the Ministry of Health before starting this study.

### 2.2. Definitions

The SBCE findings were classified according to the Saurin classification to determine their clinical significance [12]. The findings were classified as P0 when they had no clinical significance, P1 when they had an uncertain hemorrhagic potential, and P2 when they had a high relevant bleeding source (Table 1). “Diagnostic yield” was defined as the identification of a lesion that had a definite or possible pathological potential (P1 and P2). The completion of SBCE study was defined as the complete passage of the capsule through the small bowel confirmed by the visualization of the colon during the examination or the direct visualization of the excretion of the capsule from the colon by the patient.

### 2.3. Patients

The study included all adult patients who underwent SBCE for suspected small bowel pathology during the study period. All patients underwent upper and lower endoscopies before inclusion, and some underwent abdominal imaging (CT, MRI) as deemed appropriate by their referring physician. Patients with suspected or confirmed small bowel obstruction or strictures were excluded from the study until radiological imaging was performed to rule out such pathologies. Patients who were not able to swallow the capsule or were known to have severe gastroparesis had the capsule delivered endoscopically.

### 2.4. Capsule Endoscopy Procedures

The MiroCam^®^ capsules (Intromedic, Seoul, Republic of Korea) were used for all procedures. Patients were allowed to have a light meal the day before the procedure and given low-volume oral purge (2 sachets of sodium phosphate) the night before the procedure. Patients ingested the capsule in the morning and remained fasting for the next 4 h, followed by a fluid diet and slow advancement of their diet throughout the day. The recorder was disconnected and brought back to the endoscopy unit the next morning. All SBCE images were reviewed and interpreted by 2 experts (AA, AD) both with formal training in SBCE interpretation and more than 5 years of experience in reading SBCE studies. All abnormal findings were reviewed again for the purpose of this study for confirmation. The final diagnosis and Saurin classification were recorded if both reviewers agreed on the findings. In cases of suspected capsule retention (clinical features of obstruction or the capsule not reaching the cecum on recorded imaging), the patients were brought back to the hospital for clinical assessment and the performance of an abdominal X-ray. If the patient required endoscopic or surgical intervention to remove the capsule, or the capsule failed to reach the cecum by 2 weeks, capsule retention was diagnosed.

### 2.5. Outcomes

The main outcome measure was the “diagnostic yield” as defined above. Other secondary outcomes included capsule retention rate, complications, and the proportion of patients whose management was altered due to the findings of the SBCE study. In addition, baseline patient’s factors that were associated with increased odds of identifying significant pathology on SBCE were explored.

### 2.6. Statistical Analysis

Descriptive statistics were carried out and reported as mean ± standard deviation or percentage. Univariable and multivariable logistic regression analyses were performed to assess the association between baseline factors and identifying significant pathology on SBCE. The regression model was presented as odd ratios (OR) with a null value of “1”. A statistical significance threshold of *p* < 0.05 and confidence interval (CI) of 95% was adopted for all associations. All analyses were performed using STATA software version 15.1 (STATA corp. LP, College Station, TX, USA).

## 3. Results

In total, there were 210 patients that underwent SBCE examination during the study period, and none were excluded from the final analysis. The majority were males (129 patients, 61.4%) with a mean age of 57.9 (±18.5) years. Adequate bowel preparation was achieved in 88.1% of patients, and SBCE study of the small bowel was complete in 92.4% of patients. There were no cases of capsule retention or complications directly related to the procedure (Table 2).

Many of the included patients had multiple indications for SBCE. The most common indication was overt OGIB manifesting as melena or hematochezia (149 patients, 70.9%), while occult OBIG, commonly manifesting as unexplained iron deficiency anemia (IDA), accounted for 28.6% (60 patients). Other less common indications for SBCE included the presence of GI symptoms (diarrhea, weight loss), inflammatory bowel disease, and abnormal small bowel imaging (Table 2).

### 3.1. SBCE Findings

The overall diagnostic yield of SBCE was 68.1%. The most common abnormality detected was small bowel vascular lesions (84 patients, 40.0%), followed by small bowel erosions (48 patients, 22.9%), and ulcers (48 patients, 22.9%). Other small bowel pathologies identified include small bowel mucosal abnormalities (erythema, edema, fold thickening) (13.8%), mass lesions (0.5%), sub-epithelial lesions (2.9%), polyps (0.5%), and strictures (3.8%). Blood in the small bowel with or without a definite lesion was identified in 18.1% of all cases, while non-specific red spots were identified in 7.6% (Table 3).

### 3.2. Post CE Follow-Up

The CE findings altered the management in 147 patients (70.0%). Repeat endoscopy (5.4%) and colonoscopy (11.6%) were recommended in some patients due to the presence of pathology within the reach of these procedures. Push enteroscopy was recommended in 38 patients (25.9%), while deep enteroscopy was suggested in 31 patients (21.1%). Medical therapy was recommended in 53 patients (36.1%) based on the SBCE findings, usually in the form of iron replacement therapy or octreotide for small bowel vascular lesions (Table 4).

### 3.3. Saurin Classification and Predictors of Abnormal Findings

**Figure 1:**
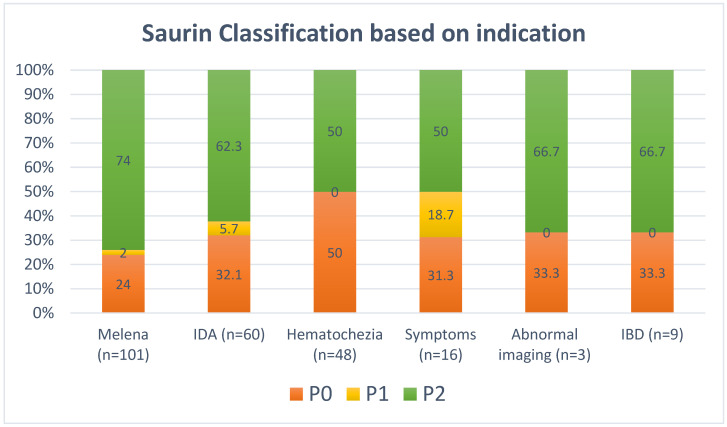
Saurin Classification Based on Indication for CE.

The majority of patients had definite significant pathological findings (Saurin class P2) on SBCE (130 patients, 61.9%), while 13 patients (6.2%) had findings of uncertain significance (Saurin class P1). The remaining patients had non-significant findings (Saurin class P0). The stratification of Saurin classification based on the indication for SBCE is shown in Figure 1. On multivariate regression analysis, melena at baseline was the only baseline factor significantly associated with increased odds of identifying significant pathology (Saurin class P2) on SBCE (OR 2.10, 1.03–4.30) (Table 5).

## 4. Discussion

Capsule endoscopy is an important diagnostic tool in the evaluation of suspected small bowel disease, and its use has been steadily increasing since its introduction over 2 decades ago. Nevertheless, optimizing patient selection remains a challenge in clinical practice due to the heterogeneity of the indications for SBCE, with several indications having low diagnostic yield, which results in patients not benefiting from this test and being likely better served with other testing modalities [13,14,15]. Our study found an overall diagnostic yield of 68.1% for SBCE to identify significant small bowel pathologies, which is similar to what has been previously reported in the literature [9,16,17]. More importantly, we were able to identify clinical factors that increase the diagnostic yield of SBCE, specifically the presence of melena as a symptom that is associated with a 2-fold increase in the odds of identifying significant small bowel pathology (OR 2.10, 1.03–4.30). This finding can assist physicians and centers performing SBCE in prioritizing patients referred with melena to undergo testing on a more urgent basis. This strategy can help to optimize the use of this limited resource while avoiding any delays in patient care. On the other hand, patients with other gastrointestinal symptoms, including abdominal pain and diarrhea, and those referred for abnormal radiological imaging had an overall lower diagnostic yield, and performing SBCE routinely for this subgroup of patients may not be indicated nor cost-effective. While other studies identified other baseline factors that were associated with an increased diagnostic yield for SBCE, including increasing age [18,19] and male gender [19], our finding of the strong association between the presence of melena and identifying significant abnormalities on SBCE is novel and should be incorporated into the decision-making when triaging referrals for SBCE. A study by Lepileur et al. identified overt OGIB (including melena and hematochezia) as an important predictor for identifying significant pathology on SBCE; this study did not discriminate between patients presenting with melena and hematochezia [20], and according to our findings, melena seems to be the more significant baseline factor associated with increased detection of pathology on SBCE.

An important indication for SBCE in clinical practice is unexplained IDA without overt GI bleeding after a negative upper and lower endoscopy (occult OGIB). A previous study by Olano et al. concluded that a lower hemoglobin level is significantly associated with identifying significant disease on SBCE among patients with IDA [19]. Even though we were not able to find a significant correlation between the presence of IDA and identifying significant pathology on SBCE, this could be related to the inclusion of a relatively small number in this subgroup (60 patients). In addition, our study included patients with different small bowel pathologies rather than focusing on patients with angiodysplasia, as was the case in the study by Olano et al. The latest European society of gastrointestinal endoscopy (ESGE) guidelines suggest performing SBCE in patients with unexplained IDA after negative bidirectional endoscopy [9], a practice that had a diagnostic yield of 62.3% in our cohort, compared to a diagnostic yield of 74% among patients with overt bleeding (i.e., melena). Therefore, it seems that even though unexplained IDA is a reasonable indication for SBCE, patients presenting with overt bleeding should be prioritized due to the higher diagnostic yield in this group.

An interesting observation in our study is the relatively high rate of identifying lesions within the reach of upper and/or lower endoscopes that were missed during the initial evaluation. Lesions in the upper GI tract were identified in 5.4%, while those in the lower GI tract were identified in 11.6%. This highlights the importance of high-quality endoscopic examination among all patients, especially those being referred for SBCE. In addition, it emphasizes the importance of careful assessment of the gastric and colonic images recorded on SBCE since they may provide valuable information. Despite the current recommendation from the ESGE to avoid second-look endoscopic examination among patients referred for SBCE [9], this practice should be individualized to each center based on the local data. In our center, all endoscopic examinations at the time of SBCE referral are reviewed carefully, and if there are any doubts regarding the quality of the initial study, we usually repeat endoscopic examinations as deemed appropriate. When lesions in the proximal small bowel that are within the reach of push enteroscopy are considered (25.9%), the overall proportion of patients with GI lesions that were within the reach of routine endoscopic procedures (endoscopy, push enteroscopy, and/or colonoscopy) was approximately 43%, further highlighting the importance of careful assessment of patients prior to performing SBCE. When a repeat endoscopic examination is deemed necessary before SBCE, consideration of push enteroscopy during the repeat endoscopic examination might be a reasonable strategy to maximize the diagnostic yield. Further studies are required to address which subgroup of patients referred for SBCE warrant repeat endoscopic evaluation before undergoing SBCE examination.

The findings of our study support the important role of SBCE in management of patients with suspected small bowel disease. Among the 210 patients referred for SBCE, the results of the SBCE altered the treatment in 70% of these patients. Among this group, 94 patients were advised to undergo endoscopic evaluation including repeat upper or lower endoscopy or deep enteroscopy, mostly to treat vascular lesions. However, some patients (n = 53) were advised to be managed conservatively with observation only or medical therapy. Some of these patients had a negative SBCE where no etiology was identified; hence, observation was considered sufficient due to the low risk of rebleeding in this subgroup. Several studies previously reported a relatively high proportion of negative SBCE among patients undergoing testing for suspected small bowel bleeding [21,22,23]. However, this lack of identification of a significant pathology on SBCE may actually have a positive impact on the patient’s outcome and can be helpful in planning further interventions for the patient. Riccioni et al. followed 207 patients with OGIB who had a negative SBCE and found a relatively low (16.4%) rate of rebleeding at 2 years follow-up [24]. Therefore, a negative SBCE can be useful in certain patients since they have a low risk of rebleeding, and further invasive investigations can be deferred. This was supported by our study where conservative treatment was suggested in a subset of patients with a negative SBCE rather than pursuing further investigations.

Our study has several strengths. All SBCE examinations were reviewed by two experts with vast experience in reading and interpreting the SBCE images, increasing the reliability of the findings. In addition, for the purpose of this study, all abnormal findings were reviewed again for confirmation, further strengthening the reliability and accuracy of the initial interpretation. The follow-up data for all patients was obtained using the electronic health system, and none of the patients were excluded due to a lack of clinical data. Even though the main focus was on patients with OGIB, the inclusion of patients referred for a variety of suspected small bowel diseases increases the generalizability of the findings of this study. One of the limitations of the study was the single-center retrospective design, which may have introduced selection and recall bias. Nevertheless, being a referral center for most SBCE in Kuwait, this increases the reliability of our findings, and as mentioned previously, accessing the electronic health care records ensured that missed data was kept to a minimum. Another limitation is the lack of data on the use of antithrombotics at baseline, which is an important factor that has been associated with increased risk of GI bleeding and more positive findings on SBCE [25,26]. Similarly, some relevant baseline patient factors, including the use of non-steroidal anti-inflammatory medications, baseline laboratory information (e.g., hemoglobin level, renal function), and some clinical symptoms (e.g., previous bleeding and surgeries) were not available, limiting the interpretation of the impact of these baseline factors on patient outcomes. Finally, we did not take into account the timing from the onset of symptoms to the performance of SBCE, a factor that might have an important influence on the detection of abnormalities in suspected small bowel bleeding [27]. Nevertheless, our findings support the higher yield of SBCE in patients presenting with melena, irrespective of the timing of the onset of their symptoms compared to other indications.

In conclusion, SBCE has a fair diagnostic yield among carefully selected patients with suspected small bowel disease coupled with an excellent safety profile. Patients referred for the investigation of overt OGIB, specifically when presenting with melena, have a higher diagnostic yield and should be prioritized for this test. Further larger multicenter studies are required to confirm this observation and assess other baseline factors that may improve the diagnostic yield of SBCE.

## Figures and Tables

**Table 1:** Saurin classification of small bowel lesions seen at SBCE.

Lesion Type	Bleeding Potential	Example
P0	None	Visible submucosal vein Diverticula without blood Nodule without mucosal breaks Erythematous patch
P1	Uncertain	Red spots Small or isolated erosions
P2	High	Angioectasia Large ulcers Tumors Varices

**Table 2:** Baseline characteristics of included patients and capsule endoscopy studies (n = 210).

Variable	Results
Gender, n (%)	
Male	129 (61.4)
Female	81 (38.6)
Age, mean (SD)	57.9 (18.5)
Indications, n (%) *	
Overt Obscure GI bleeding	149 (70.9)
Occult Obscure GI bleeding	60 (28.6)
Abnormal imaging	3 (1.4)
Inflammatory bowel disease	9 (4.3)
Other GI symptoms	16 (7.6)
Adequate bowel preparation, n (%)	185 (88.1)
Small bowel transit time, min (SD)	282.3 (134.3)
Capsule endoscopy completion, n (%)	194 (92.4)
Capsule endoscopy retention, n (%)	0 (0)
Capsule endoscopy complications, n (%)	0 (0)

* Some patients had >1 indication for capsule endoscopy.

**Table 3:** CE findings and Saurin classification *.

Variable	Results
Vascular lesion in small bowel	84 (40.0)
Small bowel erosion	48 (22.9)
Small bowel ulcer	48 (22.9)
Small bowel mucosal abnormality	29 (13.8)
Red spots in small bowel	16 (7.6)
Small bowel stricture	8 (3.8)
Small bowel submucosal lesion	6 (2.9)
Small bowel polyp	1 (0.50)
Small bowel mass	1 (0.50)
Blood in small bowel	38 (18.1)
Saurin classification **	
P0	67 (31.9)
Normal	54 (25.7)
Erythematous patch only	13 (6.2)
P1	13 (6.2)
Red spot	10 (4.8)
Erosion with erythematous patch	3 (1.4)
P2	130 (61.9)
Vascular lesion	84 (40.0)
Small bowel ulcer	26 (12.4)
Small bowel stricture	8 (3.8)
Small bowel mass/polyp	8 (3.8)
Blood in small bowel	4 (1.9)
Altered management	147 (70)

* Some patients had >1 abnormality detected on SBCE. ** Saurin classification based on the most significant findings on SBCE.

**Table 4:** Post-capsule endoscopy recommendations.

Post-CE Procedures	Results
Endoscopy	8 (5.4)
Colonoscopy	17 (11.6)
Push enteroscopy	38 (25.9)
Double balloon enteroscopy	31 (21.1)
Medical treatment	53 (36.1)

**Table 5:** Logistic regression analysis for odds of finding significant pathology on capsule endoscopy (Saurin classification P2) based on patient characteristics and presentation.

Variable	Univariate		Multivariate	
	Odd Ratio (95% Confidence Interval)	*p*-Value	Odd Ratio (95% Confidence Interval)	*p*-Value
Older age (Age ≥ 60 years)	1.52 (0.88–2.73)	0.2	1.47 (0.83–2.58)	0.2
Male gender	1.40 (0.80–2.48)	0.3	1.37 (0.78–2.43)	0.3
Melena	2.25 (1.10–4.42)	0.04	2.10 (1.03–4.30)	0.04
Fresh blood per rectum	0.63 (0.20–2.23)	0.4	0.60 (0.17–2.17)	0.4
Iron deficiency anemia	1.10 (0.50–2.1)	0.9	1.04 (0.54–1.98)	0.9
Abnormal radiological imaging	1.34 (0.10–14.58)	0.8	1.29 (0.11–14.55)	0.8

## Data Availability

The original contributions presented in the study are included in the article, further inquiries can be directed to the corresponding author.

## Abbreviations

CT	Computed tomography
CI	Confidence intervals
ESGE	European Society of Gastrointestinal Endoscopy
GI	Gastrointestinal
IDA	Iron deficiency anemia
SBCE	Small bowel capsule endoscopy
OGIB	Obscure gastrointestinal bleeding
